# Is the Postoperative Outcome after Intraarticular Distal Radius Fracture Influenced by Age and Gender? A PROM Study

**DOI:** 10.3390/jcm12031202

**Published:** 2023-02-02

**Authors:** Francesca Von Matthey, Johannes Rüdiger Rammensee, Peter Biberthaler, Helen Abel

**Affiliations:** Department of Trauma Surgery, Klinikum Rechts der Isar, Technische Universität München, 80333 Munich, Germany

**Keywords:** distal radius fracture, gender health gap, PROM, MWQ, osteoporosis

## Abstract

Background: Although the most common fracture of the human body, so far, only few data concerning gender-specific outcomes after distal radius fracture exist. Typically, elderly women suffer from DRF due to a low-energy fall because of osteoporosis. The aim of this study was to analyze the outcome after a surgically treated intraarticular DRF with the help of patient-reported outcome measurement (PROM) and with special regard to gender and age impact. Patients and methods: It is a retrospective case-control study in which all patients with an intraarticular DRF were enrolled. The control group was composed of healthy volunteers. Munich Wrist Questionnaire (MWQ) was used as Patient Related Outcome Measurement (PROM) for analyzing the outcome. Moreover, age, gender, handedness, fracture classification and follow-up interval were detected. The functional outcome of the fracture group was compared to a healthy control group. Analyses of gender, age and handedness-specific results compared to the healthy control groups were performed as well. Results: 197 patients with distal radius fracture could be enrolled in the study (134 were female and 63 were male). Women (mean age 62 years) were significantly older than men (mean age 50 years). The control group comprised 110 healthy subjects, 71 females (mean age 56 years) and 39 males (mean age 53 years). The whole fracture group had significantly lower MWQ scores compared to the control group (*p* < 0.001). The male fracture group (90.6 ± 12.4) and the female fracture group (90.8 ± 11.4) had a significantly worse outcome compared to the corresponding control group (*p* < 0.001 male and *p* = 0.034 female). Although significantly younger, the male patients had a similar outcome compared to the female patients. Discussion: Even elderly patients can reach the preoperative level after operative treatment of an intraarticular distal radius fracture. Although significantly younger than the female patients, men have significantly worse functional outcomes compared to their control and cannot perform better than the significantly older female patients. Gender might influence the outcome as well; however, age seems to have a greater impact on the outcome than gender.

## 1. Introduction

Distal radius fractures (DRF) are the most common fractures of the human body. There are two age groups that are affected the most. These are young men and older women. The first is due to sports accidents and a fall on the outstretched hand, while the second is due to suffering from progressive osteopenia or osteoporosis because of low-energy trauma (a rather minor fall). However, the majority of the patients suffering from a distal radius fracture, are older women. These women very often have a history of recurrent falls, but they are in most other aspects of general health and lifestyle very similar to women who do not fracture the wrist. However, the fractures contribute to a significant functional decline in these women, as defined by a worsening ability to prepare meals, perform heavy housekeeping, go shopping or get out of a car [1,2].

Several studies within the last few decades have analyzed the outcome following an operative or conservative treatment of distal radius fractures. Special attention has been turned to the question of whether surgical or conservative treatment is superior. This question needs to be differentiated from fracture type, patient age and bone quality, respectively. However, most studies have good results after osteosynthesis [3,4,5,6]. A randomized controlled trial showed in the long run no difference between conservative and operative treatment but significantly better grip strength, DASH and PRWE scores as well as more complications after operative treatment [3,7,8]. Nevertheless, recovery after surgical treatment of a DRF is quicker and those patients are faster back to daily business, which is important, especially for elderly patients that live alone [3,9,10]. However, possible severe complications after surgery such as nervus medianus affection, carpal tunnel syndrome, peri-implant fractures and CRPS should be taken into consideration [11,12,13].

However, although the DRF is a typical fracture of elderly women, so far, no study concerning gender-specific outcomes exists. Despite the higher fracture risk in postmenopausal women, older men tend to have worse outcomes in general after fracture and poorer treatment rates [14,15]. Concerning other fractures, such as hip fractures, studies could even prove a higher mortality rate in men after hip fractures than in women [16].

This study aimed to analyze the outcome after surgically treated intraarticular distal radius fracture with the help of PROM and to analyze whether there is a gender- or age-specific outcome.

## 2. Patients and Methods

Our study is a retrospective case-control study, which was reviewed by the Ethics Committee 409/15s of our hospital.

### 2.1. Patients

All intraarticular distal radius fractures (type B and C according to the AO classification), which were treated with plate osteosynthesis in our clinic between 2006 and 2016, were included in our study.

Exclusion criteria were lack of capacity/absence of consent, age under 18 years, pathological fracture, polytrauma, pre-existing injuries to the ipsilateral limb and all distal radius fractures without joint involvement.

### 2.2. Control Group

Healthy volunteers served as the control group. Subjects were excluded from the control group if they had already suffered an upper limb injury, had pre-existing conditions affecting wrist function or were unable to consent to the study.

### 2.3. Surgical Therapy

All patients were treated with a standardized surgical procedure. This included the Henry volar approach and volar plate osteosynthesis using a stable-angle plate (Depuy Synthes, Medartis, etc.).

Patients received physiotherapy and radiological and clinical follow-up as part of a standardized postoperative regimen.

### 2.4. Target Demographic Parameters

Age, gender and fracture type according to AO classification were documented, as well as the interval between surgical treatment and follow-up examination.

### 2.5. Examination of Wrist Function Using PROM

Wrist functions, both of the patients and the control group, were evaluated using the Munich Wrist Questionnaire (MWQ). 

The MWQ is a validated questionnaire on wrist function and was developed as a Patient Reporting Outcome Measurement and is used for patient self-evaluation. The questionnaire contains both objective and subjective factors on wrist function. The result is expressed as a percentage, with 100% corresponding to full wrist function [17].

### 2.6. Data Analysis

Epidemiological and demographic data such as sex, age and follow-up period were analyzed, which were reported in Mean ± standard deviation (M ± SD) or as a percentage.

Quantitative data analysis of wrist function was performed by SIGMASTAT and SIGMAPLOT using the t-test or Mann–Whitney U-test. Moreover, multiple linear regression analysis was performed between age (dependent variable) and MWQ Score (independent variable).

In the first step, we examined the entire collective in comparison to the control group with regard to function and age. In a further step, the total collective was sorted according to gender and the gender-specific analysis with regard to age and function was carried out. For this purpose, an additional distinction was made between young (<65 years) and old (>64 years) patients. Finally, the groups were sorted according to the dominant hand and the function of the right dominant hand was examined, taking age and gender into account. A comparison was made between the fracture group and the control group as well as a direct comparison between female and male patients.

The function of these subgroups was analyzed using the *t*-test or the Mann–Whitney U-test for pairwise comparison of not normally distributed data. The significance level was assumed to be *p*-value < 0.05.

## 3. Results

### 3.1. Patient Group

**Figure 1:**
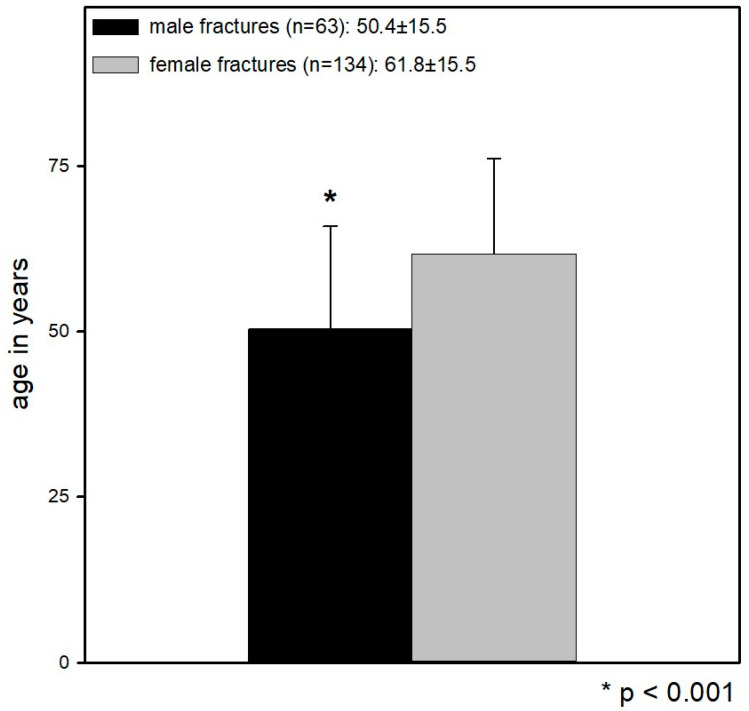
Mean age of the female patients was 62 years (61.8 ± 15.5) (M ± SD) and of the male patients 50 years (50.4 ± 15.5) (M ± SD). Male patients were significantly younger than female patients (*p* < 0.001).

The patient group comprised 197 patients with distal radius fracture and no other injuries or previous injuries to the affected extremity, of which 134 were female and 63 male patients with a mean age of 62 years (61.8 ± 15.5) (M ± SD) in the female and 50 years (50.4 ± 15.5) (M ± SD) in the male patients. Male patients were significantly younger than female patients (*p* < 0.001) (Figure 1).

There were 67 females and 50 males in the <65 years subgroup and 67 females and 13 males in the >64 years group.

Of the female patients, the right hand was dominant in 124 cases and the left hand in 10 cases. In the male patients, the right hand was dominant in 55 cases and the left hand in 8 cases. Patients who stated that they had no dominant wrist function were excluded from this study.

25 patients had a fracture type B according to the AO classification and 172 patients had a Type C fracture. 

The average follow-up was 67 (67.1 ± 31.8) months.

### 3.2. Control Group

Our control group comprised 110 healthy subjects, with no previous upper extremity disease. Both right and left wrist functions were evaluated. The control group included 71 females and 39 males, with a mean age of 56 years (55.8 ± 19.8) (M ± SD) for females and 53 years (52.7 ± 16.4) for males.

Notably, 40 female and 34 male subjects were <65 years old, and 31 female and 5 male subjects were >64 years old.

In the female subjects, the right hand was dominant in 67 cases and the left hand in 4 cases. In the male subjects, the right hand was dominant in 31 cases and the left hand in 8 cases. (Table 1).

### 3.3. Function (Results of the MWQ)

The function of both the control group and the fracture group was evaluated using the MWQ. Achieving 100% in the MWQ meant complete wrist function and freedom from symptoms.

**Figure 2:**
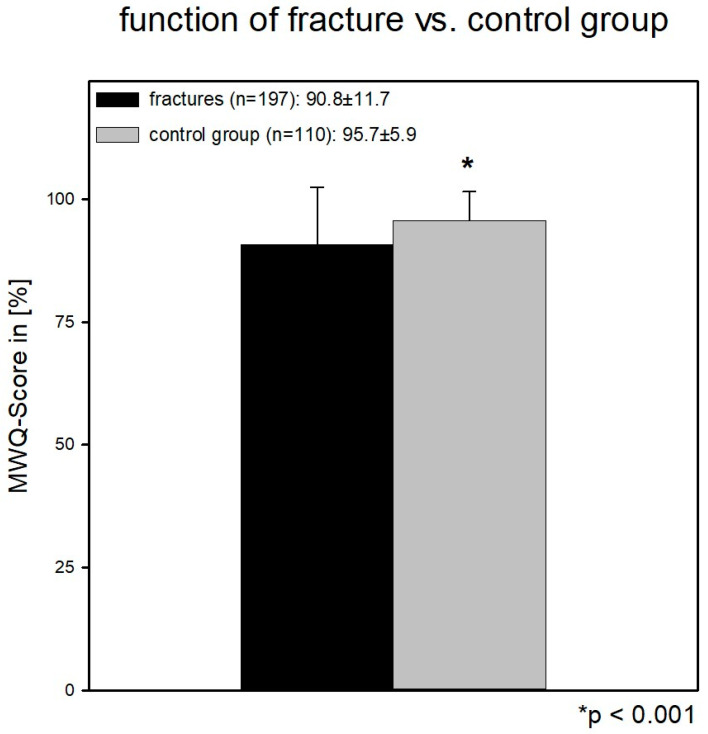
Function of the patients vs. the control group: the control group showed significantly better wrist function than the patient group (95.7 ± 5.9 control and 90.8 ± 11.7 fracture) (*p* < 0.001).

Regarding the whole study collective, the control group showed significantly better wrist function than the patient group (95.7 ± 5.9 control and 90.8 ± 11.7 fracture) (*p* < 0.001). Figure 2.

**Figure 3:**
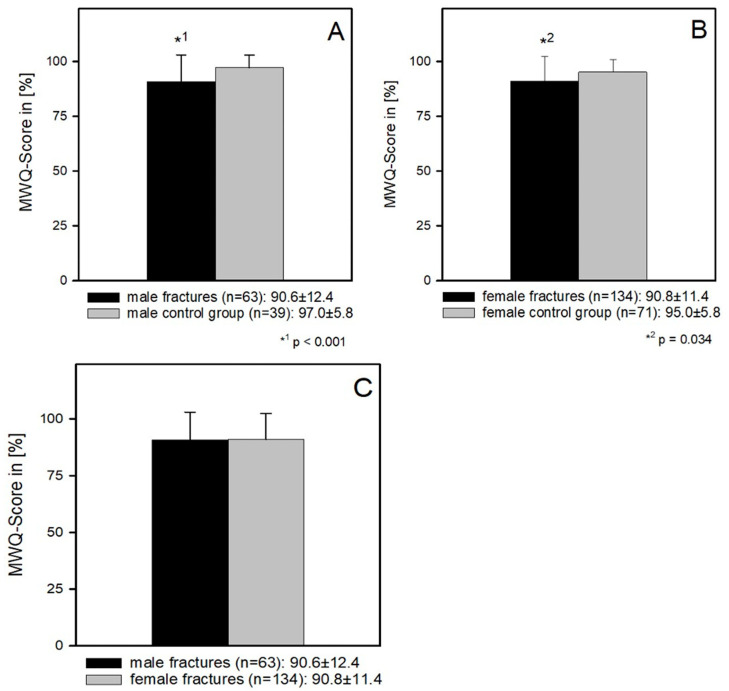
(**A**) Male patients had a significantly worse outcome compared to the control group MWQ (97 ± 5.8 and 90.6 ± 12.4) (*p* < 0.001). (**B**) The same result was found in the comparison between the female patients and the female control group (90.8 ± 11.4 and 95 ± 5.8) (*p* = 0.034). (**C**) There was no difference in function between female and male patients (90.8 ± 11.4 and 90.6 ± 12.4) (*p* = 0.85).

Taking gender into account, the comparison between the male control group and the male patients also showed a significantly better functional result in the control group MWQ (97 ± 5.8 and 90.6 ± 12.4) (*p* < 0.001) Figure 3A. The same result was found in the comparison between the female patients and the female control group (90.8 ± 11.4 and 95 ± 5.8) (*p* = 0.034) Figure 3B. There was no difference in function between female and male patients (90.8 ± 11.4 and 90.6 ± 12.4) (*p* = 0.85) (Figure 3C).

**Figure 4:**
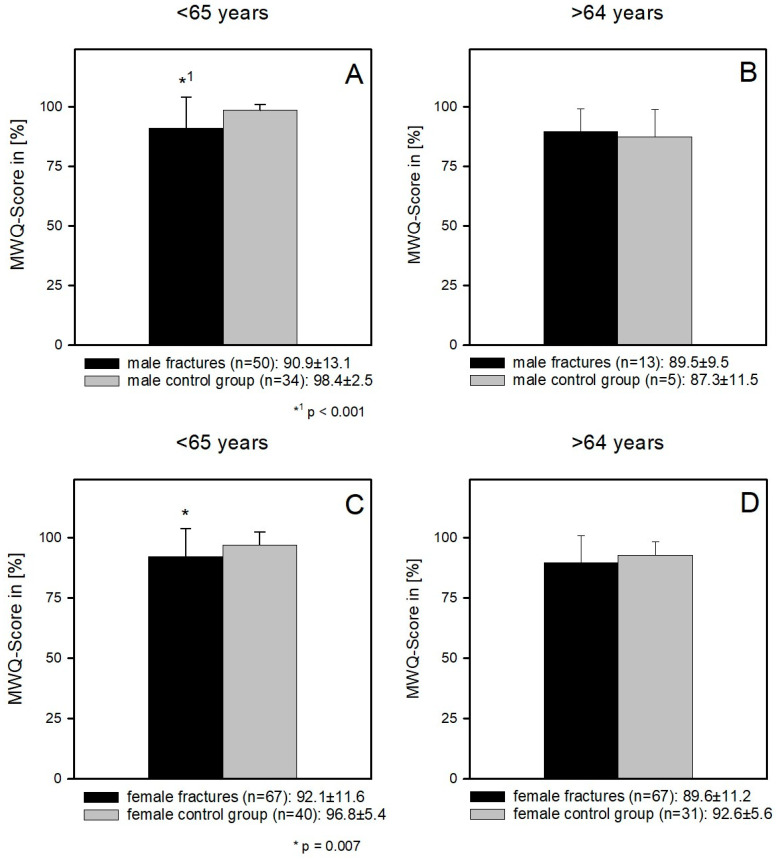
(**A**) Young male patients (<65 years) had a significantly worse functional outcome compared to the corresponding control group (90.9 ± 13.1 and 98.4 ± 2.5) (*p* < 0.001). (**B**) Elderly male patients (>64 years) could reach the functional level of their corresponding control group (89.5 ± 9.5 and 87.3 ± 11.5) (*p* = 0.7). (**C**) young female patients (<65 years) had a significantly worse functional outcome compared to the corresponding control group (92.1 ± 11.6 and 96.8 ± 5.4) (*p* = 0.007). (**D**) Elderly female patients (>64 years) could reach the functional level of the corresponding control group (89.6 ± 11.2 and 92.6 ± 5.6) (*p* = 0.86).

In order to evaluate the influence of age in consideration of gender, further analysis was carried out in which female and male patients aged <65 years and >64 years, respectively, were compared with the control group (Figure 4A–D).

The comparison of young female patients (<65 years) and the corresponding control groups showed a significantly better wrist function in the control group (92.1 ± 11.6 and 96.8 ± 5.4) (*p* = 0.007). In the female patients >64 years of age, there was no postoperative functional difference to the control group (89.6 ± 11.2 and 92.6 ± 5.6) (*p* = 0.86) (Figure 4C,D).

The situation was similar in the male patients. The comparison of the <65-year-old male patients with the corresponding control group showed a significantly worse postoperative function of the fracture group (90.9 ± 13.1 and 98.4 ± 2.5) (*p* < 0.001). As well as in the group of >64-year-old female patients, there was also no significant functional difference when comparing the older male patients with their control group (89.5 ± 9.5 and 87.3 ± 11.5) (*p* = 0.7) (Figure 4A,B).

**Figure 5:**
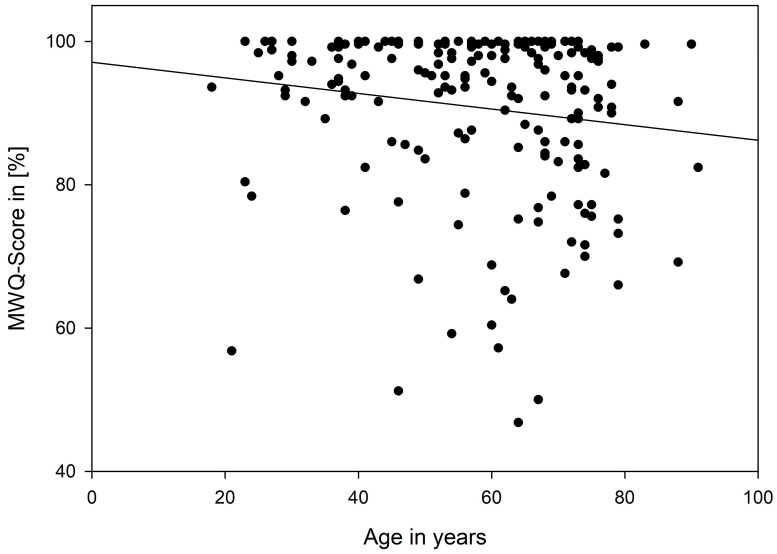
Multiple linear regression analysis of the fracture collective.

Regarding the correlation between age and MWQ in the fracture collective, the multiple regression analysis showed an increasing MWQ Score with increasing age (*p* = 0.041) Figure 5.

**Figure 6:**
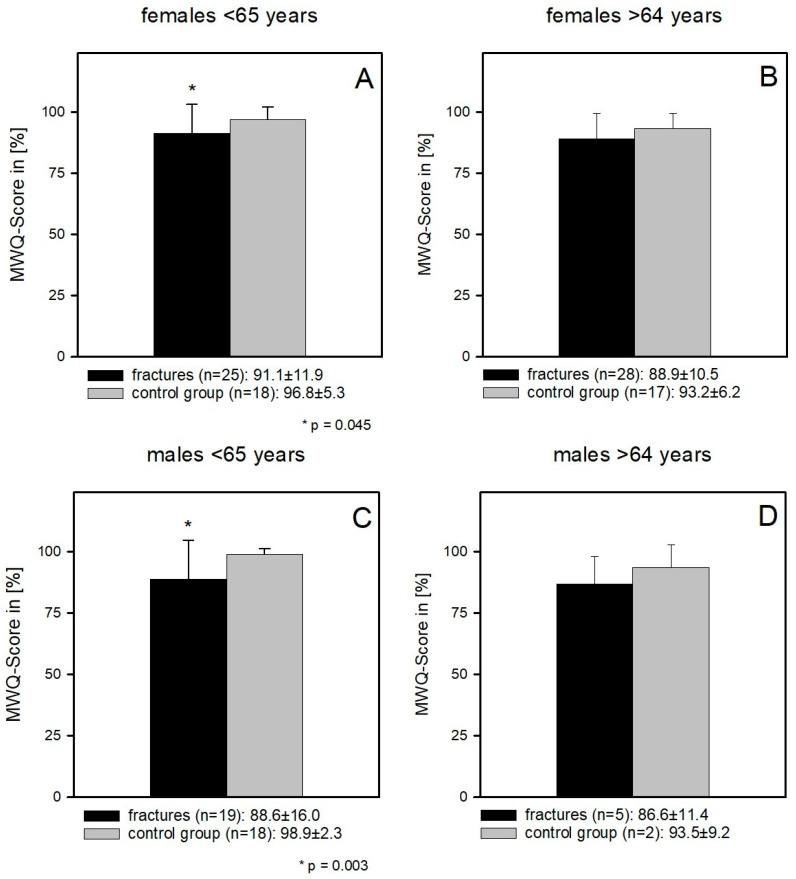
(**A**) Female patients < 65 years, in whom the right, dominant wrist was fractured had a significantly worse functional outcome compared to the corresponding control group (91.1 ± 11.9 and 96.8 ± 5.3; *p* = 0.045) (*p* = 0.045). (**B**) Female right-handed dominant patients >64 years could reach the functional level of the corresponding control group (88.9 ± 10.5 and 93.2 ± 6.2) (*p* = 0.44). Figure 6B. (**C**) Male patients < 65 years, in whom the right, dominant wrist was fractured had a significantly worse functional outcome compared to the corresponding control group (<65 years: 88.6 ± 16 and 98.9 ± 2.3; *p* = 0.003). (**D**) Male right-handed dominant patients of >64 years could reach the functional level of the corresponding control group (>64 years: 86.6 ± 11.4 and 93.5 ± 9.2; *p* = 0.49).

Female patients < 65 years, in whom the right, dominant wrist was fractured, had a significantly worse functional outcome compared to the corresponding control group (91.1 ± 11.9 and 96.8 ± 5.3; *p* = 0.045) (*p* = 0.045) (Figure 6A). In contrast, the female right-handed dominant patients >64 years could reach the functional level of the corresponding control group (88.9 ± 10.5 and 93.2 ± 6.2) (*p* = 0.44) (Figure 6B).

The male population had similar results (<65 years: 88.6 ± 16 and 98.9 ± 2.3; *p* = 0.003) and (>64 years: 86.6 ± 11.4 and 93.5 ± 9.2; *p* = 0.49) (Figure 6C,D).

## 4. Discussion

Numerous studies have been published concerning distal radius fracture, optimal treatment options, the outcome, epidemiology and etiology. Being the most common fracture of the human body, it is astonishing that so far, only a few data concerning gender-specific outcomes exist. It is a fact that especially elderly women suffering from osteopenia or osteoporosis are likely to sustain a distal radius fracture after low-energy trauma. Although several authors could show a gender-specific outcome for hip fractures, for example, which is more favorable for women than for men [16,18], no publication has analyzed the gender-specific outcome for distal radius fractures. The background is similar to hip fractures as the most important risk factor for DRF is poor bone quality, as it can be found in osteoporosis. However, studies on hip fractures show that the outcomes differ significantly between men and women, and moreover, the excess annual mortality after hip fracture is even higher in men than in women [14,15,16]. A meta-analysis of 24 studies, including data from 578,436 women and 154,276 men, estimated the excess mortality risk after hip fracture for both men and women. Excess annual mortality persists over time for both women and men, but at any given age, excess annual mortality after hip fracture is higher in men than in women [16].

We took this as an opportunity to analyze the influence of gender and age in distal radius fractures in a retrospective study. Since the distal radius fracture, such as the coxal femur fracture, is regarded as an indicator fracture for osteoporosis and accordingly predominantly older women are affected, we wanted to investigate whether this is also reflected in the postoperative outcome.

In accordance with the results in the current literature, more women than men were affected by the distal radius fracture in our collective, and the female patients were on average significantly older than the men.

The postoperative wrist function was examined using PROM, but there was no significant difference in the postoperative outcome between female and male patients with regard to wrist function. Overall, all patients showed a very good functional outcome after surgical treatment of a distal radius fracture.

The young patients showed significantly worse postoperative wrist function than the healthy controls, irrespective of gender. A direct comparison of the postoperative wrist function of young female and young male patients also showed no difference. This is most likely due to the naturally good wrist function at a young age and the high functional demands of young patients. Although there was no gender-specific functional difference in the postoperative wrist function of the old patients, we were able to show that the old patients achieved the functional level of the control group again, regardless of gender. There was no postoperative difference in wrist function between the elderly women and the corresponding control group, nor was there any difference between the elderly men and their control group. The direct comparison between the postoperative function of the female patients and the male patients also showed no significant difference. However, we would like to discuss the statement that there is no gender-specific difference with regard to the postoperative function of distal radius fractures, as the overall group of male patients was significantly younger than the female patients, but could reach only the same functional level as the significantly older female patients did. It remains unclear whether this was due to the relatively small number of male patients in our study or actually is an indication of a gender-specific difference, as is also the case with coxal femur fractures, for example [14,15,16].

However, the influence of age on the postoperative outcome is clearly debatable. Concerning both, the male and the female fracture group older than 64 years and younger than 65 years had similar results regarding the postoperative outcome. The postoperative function was very good overall, but the difference between the young patients and the control group was significant, whereas the old patients reached the functional level of the control group, irrespective of gender. It can be concluded that older patients benefit from the surgical treatment of a distal radius fracture, as at least the female patients can regain preoperative functional levels postoperatively.

Studies could show differences in the outcome after DRF between dominant and non-dominant wrists. Wollstein et al. performed sensorimotor testing after surgical treatment of DRF and compared the results of the dominant and non-dominant wrist. They could find differences in 2 of 9 tests of sensibility for each time period [19].

Hosokawa et al. could show that Patients who sustained a dominant wrist injury were likely to report greater functional impairment, such as in opening doors, cutting meat and vegetables, pouring liquid from a pitcher, lifting pots and pans, wiping the buttocks, turning a key, arising from a chair using support, washing floors and walls, opening and closing a faucet, and experiencing morning and evening stiffness, than those who sustained a non-dominant wrist injury. They stated that outcome studies for the treatment of DRF should consider hand dominance [20].

However, these studies dealing with the outcome after dominant and non-dominant wrist injuries are hardly comparable because the analyzed parameters and the used scoring systems (DASH vs. QuickDASH vs. MWQ) are different.

Gender-specific medicine and therapy is a field of growing importance. More and more studies from all fields of medicine are being conducted. There is, for example, much evidence suggesting that gender is an important factor in the modulation of pain. Literature data strongly suggest that men and women differ in their responses to pain [18].

Nevertheless, the influence of gender on the postoperative outcome after distal radius fracture has not yet been clarified definitively.

Referring to this, more studies are required to analyze its importance.

In addition to the overall low number of patients and the heterogeneous follow-up period, another limitation of our study is the relatively small number of male patients (female n = 134 vs. male n = 63), as well as the younger average age of the male patients. However, this mirrors reality, as most of the elderly patients suffering from distal radius fractures are women due to osteoporosis [1,21,22]. Moreover, another limitation of the study is the retrospective study design, as this implies several potential biases.

## 5. Conclusions

Results after operative treatment of intraarticular distal radius fractures are very good, even in elderly patients. Regarding the postoperative functional outcome, elderly women could reach the functional level of healthy controls. Men had a similar function, but they were significantly younger, therefore raising the expectation of having a better function of the wrist. Even if the patient number is too low for drawing any major conclusions regarding a gender-specific influence on the outcome, this study points out that gender is an important topic in modern medicine. Nevertheless, age seems to have a greater impact on the outcomes than gender.

## Figures and Tables

**Table 1:** Baseline Characteristics of the study groups.

Complete Study Group Gender	Complete Study Group Age [Years]	Complete Study Group Fracture Type (AO Classification)	Complete Study Group Follow-Up [Months]	Subgroup <65 Years [n]	Subgroup >64 Years [n]	Subgroup Dominant Right Hand [n]	Subgroup Dominant Left Hand [n]
female n = 134	61.8 ± 15.5	B fracture n = 25	67 (67.1 ± 31.8) months	female n = 67	female n = 67	female n = 124	female n = 10
male n = 63	50.4 ± 15.5	C fracture n = 172		male n = 50	male n = 13	male n = 55	male n = 8

## Data Availability

The data presented in this study are available on request from the corresponding author. The data are not publicly available due to ethical reasons.
